# Deciphering structural rearrangements during transport process in the bacterial transporter GltPh, homolog to mammalian glutamate transporter

**DOI:** 10.1186/2050-6511-13-S1-A57

**Published:** 2012-09-17

**Authors:** SanthoshKannan Venkatesan, Azmat Sohail, Walter Sandtner, Thomas Stockner, Gerhard F Ecker, Harald H Sitte

**Affiliations:** 1 Institute of Pharmacology, Center for Physiology and Pharmacology, Medical University Vienna, 1090 Vienna, Austria; 2 Department of Medicinal Chemistry, University of Vienna, 1090 Vienna, Austria

## Background

Glutamate transporters are integral membrane proteins that catalyze the concentrative uptake of glutamate from the synapse by harnessing pre-existing ion gradients. In the central nervous system glutamate transporters are essential for normal development and function; they also are implicated in stroke, epilepsy and neurodegenerative diseases. The crystal structure of a eukaryotic glutamate transporter homologue from *Pyrococcus horikoshii*, is available at various conformations providing a structural framework for the determination of substrate and inhibitor binding to the transporter. In this study we aim to measure structural changes upon transport using lanthanide resonance energy transfer (LRET).

## Methods

Site-directed mutagenesis was employed to insert genetically encoded lanthanide binding tags (LBT) into the the protein to perform LRET measurements. Thus generated LBT mutants were expressed and purified, and the functionality of the mutants was assessed by radioligand binding assay.

## Results

Models for insertion of LBT were derived from the available crystal structures of the transporter. The wild-type and mutant proteins were expressed and purified using affinity column chromatography. Donor decay signals were recorded for LBT insertion mutants to confirm the insertion of tags. Furthermore, radioligand binding assays were performed with the mutants and they were found to be functional.

## Conclusions

Taken together these mutants serve as the starting point to probe the conformational changes that were observed in previously solved crystal structures in reconstituted proteoliposomes. This could help us to integrate the structure-function relationship in the mammalian counterparts.

